# Edge Computing, IoT and Social Computing in Smart Energy Scenarios

**DOI:** 10.3390/s19153353

**Published:** 2019-07-31

**Authors:** Inés Sittón-Candanedo, Ricardo S. Alonso, Óscar García, Lilia Muñoz, Sara Rodríguez-González

**Affiliations:** 1 BISITE Research Group, University of Salamanca, Edificio Multiusos I+D+i, Calle Espejo 2, 37007 Salamanca, Spain; 2 Grupo GITCE, Universidad Tecnológica de Panamá, Panama 0801, Panama

**Keywords:** internet of things, edge computing, distributed ledger technologies, social computing, smart energy

## Abstract

The Internet of Things (IoT) has become one of the most widely research paradigms, having received much attention from the research community in the last few years. IoT is the paradigm that creates an internet-connected world, where all the everyday objects capture data from our environment and adapt it to our needs. However, the implementation of IoT is a challenging task and all the implementation scenarios require the use of different technologies and the emergence of new ones, such as Edge Computing (EC). EC allows for more secure and efficient data processing in real time, achieving better performance and results. Energy efficiency is one of the most interesting IoT scenarios. In this scenario sensors, actuators and smart devices interact to generate a large volume of data associated with energy consumption. This work proposes the use of an Edge-IoT platform and a Social Computing framework to build a system aimed to smart energy efficiency in a public building scenario. The system has been evaluated in a public building and the results make evident the notable benefits that come from applying Edge Computing to both energy efficiency scenarios and the framework itself. Those benefits included reduced data transfer from the IoT-Edge to the Cloud and reduced Cloud, computing and network resource costs.

## 1. Introduction

Cloud Computing has become a means of consolidating computing functions, storage and network management centrally [1]. Unfortunately, architectures based exclusively on a centralized cloud experience many difficulties because they cannot meet the requirements of the Internet of Things (IoT) paradigm, of current mobile Internet applications, nor can they support the the massive volume of data these applications generate. The users of IoT applications look for real-time response; such features add more burden to the networks, resulting in increased latency as well as storage and high bandwidth costs [2]. Other features that an IoT cloud architecture cannot easily deal with include [3]:

- security and integrity of data coming from edge devices;
- IoT devices heterogeneity (sensors, actuators, smartphones, tablets, smart bracelets, laptops, etc.) with limited storage and processing resources;
- uninterrupted, real-time service requests and responses.

These characteristics have been the motivation behind research which proposed Edge Computing as an alternative means of processing and filtering big data at the edge of the network before transmitting it to the cloud; the benefits of this include reduced bandwidth costs, storage and energy consumption [4]. Therefore, by integrating an IoT-based Edge Computing architecture, applications and services provide users with faster, more efficient and secure responses [3].

Smart energy is one of the scenarios in which IoT is applied to achieve efficient energy use; this topic has been discussed in more detail in [5]. Countries, industries, universities have developed research in energy efficiency to obtain applications capable of controlling, measuring and managing energy consumption in buildings, grids, and households. Furthermore, countless research has provided new hardware solutions (sensors, controllers or smart meters) [6]. Despite the efforts done to make users understand the importance of reducing energy consumption in public spaces, work environments, or at home, this problem continues and it is necessary to find a solution to it [7,8]. The uncontrolled consumption and waste of energy is most noticeable in public spaces; there are several reasons for this: there are many different users with different preferences/comfort requirements, very old buildings and the lack of resources for the implementation of new technology that would adapt to their infrastructure [9].

Several authors have proposed solutions to motivate, educate and raise awareness among users about the importance of smart energy consumption [9,10]. In this regard, serious games are one of the solutions proposed to improve user behaviour and these have been presented and analyzed in numerous research [11,12]. Although there is a variety of proposals, most of them only contemplate the use of serious games in homes [13], and they do not incorporate features such as sensor technologies [14], identification and location of users in real-time, or IoT devices that improve user interaction [15].

An interesting proposal has been presented by García et al. [9], where the CAFCLA framework (Context-Aware Framework for Collaborative Learning Activities) has been used to implement a serious game based on social computing in an office environment. The main objective of the game proposed by García et al. [9] is to change the behaviour of users by acquiring good energy consumption habits in a natural and progressive way. This research analyzed the CAFCLA framework and its weaknesses in the face of a large volume of data generated by users and IoT devices. In this context, CAFCLA has been integrated with an Edge Computing architecture, with the aim of conducting a case study in a public building and evaluating its performance. The public building in which this case study has been conducted was the laboratory of the BISITE Research Group of the University of Salamanca (Spain).

The rest of the paper is organized as follows: Section 2 presents a review of the state of the art of Edge Computing, IoT technologies and Smart Energy scenarios; it also addresses the research works in which IoT has been incorporated in Smart Grids, domestic, commercial and public environments. Section 3 describes GECA, a Global Edge Computing Architecture, and how it can be integrated with CAFCLA to improve this framework. Section 4 details a use case and describes the experiment and its results. Finally, Section 5 draws the main conclusions and describes future lines of research.

## 2. Edge Computing, IoT and Smart Energy

**Figure 1:**
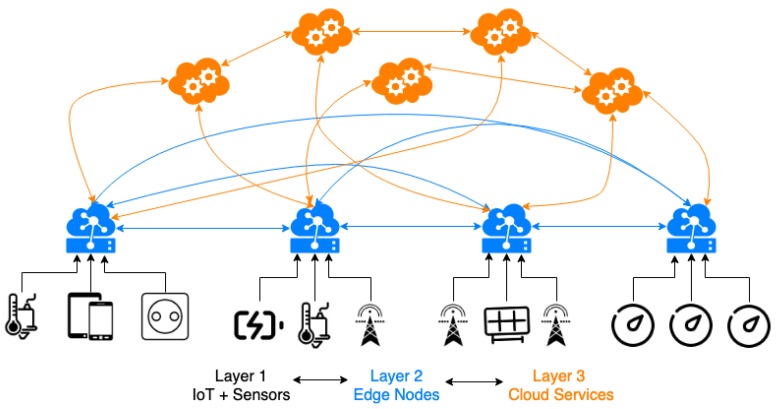
Three-layer Edge Computing basic architecture.

Over the last few years, there has been a great increase solutions that decentralize communications, data collection and processing, moving all those tasks to the edge [16]. This trend has led the emergence of the Edge Computing paradigm [17,18], whose basis is enabling technologies that perform computation at the edge of the network [17]. In this way, computing and network resources (edges) are closer to the source of the data than to the cloud data centers [17]. Edge Computing allows for improving the performance of computer systems by lowering latency, reducing the cost of resources and increasing responsiveness, scalability, reliability, security or privacy [19]. The Internet of the Things (IoT) is a well-known technology and a broad research topic that has become a reference for data collection and processing systems [20]. IoT systems are formed by multiple and heterogeneous devices (sensors, vehicles, machinery, appliances, meters, etc.) connected through different communication protocols [21]. Multiple disciplines benefit form IoT solutions such as Industry 4.0 [22,23], energy efficiency [24,25], smart homes [26,27], smart cities [28,29] or healthcare [30]. The management of IoT networks is challenging because of the heterogeneity of its resources, creating difficulties in communication protocols, real-time processes, data management, big data storage, security or privacy. In this regard, Edge Computing architectures offer a solution to IoT infrastructures because they are capable of managing the heterogeneous data generated by IoT devices [3,31]. Figure 1 gives an example of a basic three-layered Edge Computing architecture [32]:

- Layer 1—IoT + Sensors: This layer includes IoT devices (sensors, smart meters, smart plugs, etc.) as well as users. The first layer is responsible for the ingestion of data and the operations involved.
- Layer 2—Edge Nodes: The second layer is formed by Edge nodes. These nodes are responsible of data processing, routing and computing operations.
- Layer 3—Cloud Services: This layer is formed by multiple cloud services with higher computational requirements. This layer is responsible for Data Analytics, Artificial Intelligence, Machine Learning, or visualization, among other tasks.

As mentioned above, smart energy environments can benefit from IoT approaches. Monitoring energy consumption and other parameters allows for a 40% reduction of energy consumption [24]. Sensors and intelligent devices have transformed the energy industry by allowing to get a deeper understanding of how energy is spent and what factors influence its consumption. There are a large number of proposals and approaches in the literature that address this problem, although they all have different objectives, such as achieving better efficiency and resource management [33], reducing carbon emissions and costs [34], improving Demand Response (DR) systems [35], or improving knowledge and system automation by data correlation (such as temperature, humidity, luminosity or other sensors, weather information, user, segment and building profiles, etc.) [36]. Due to the wide variety of the proposed solutions, and above all, the multiple application areas, the state of the art presented here includes the most outstanding works in three different areas, Smart Grids, Industrial and commercial environments and Domestic environments.

### 2.1. Smart Grids

Energy distribution networks have great potential in terms of data analysis. The optimization of energy production and demand poses a great challenge for researchers. As a result, we find numerous works in the state of the art that address those topics. There is a rise in the use of renewable energy sources and there is an increasing number of energy production and self-consumption solutions for homes and businesses. Many research works have focused on the importance of reducing energy loss and how this contributes to energy efficiency of the system and, therefore, in the price of energy [37]. In this sense, the implementation of Smart Grids must be addressed from different perspectives, such as Distributed Generation (DG) [38] or Real-Time Pricing (RTP) programs [39].

Solutions offer different approaches to the optimization of Demand Response [35,40,41]. One of the main problems to be solved is the optimization of a distributed model that encourages as many users as possible to participate in Demand Response programs. Yaghmaee et al. [14] present a cloud-based demand response model that deals with communication networks and message management. This model has an Edge Computing approach; it uses cloud services in order to optimize the communication between the different stakeholders. However, the design does not provide any security to the communications, since it uses UDP. Furthermore, the management of edge nodes or the inclusion of sensors that optimize the collection of information are not considered by Yaghmaee et al. [14]. All these aspects are covered in our proposal because it combines the CAFCLA framework and the GECA architecture.

Moreover, the authors of Naranjo et al. [42] affirm that the computational requirements of Smart Grids may be moved to the edge of the network. In connection to this statement, their research proposes the deployment of SmartLocalGrids (SLG) as a communication paradigm between microgrids. This solution makes it possible for the nodes to collect data and transport it to a remote cloud where an in-depth analysis is carried out, and in their work Naranjo et al. [42] include an analysis of how the support of the entire infrastructure should be managed with the model they present. Similarly, the article presents a system that is compatible with IoT models. However, this proposal also has some security and reliability limitations. In this regard, it is worth mentioning that, by using the GECA architecture in our proposal, both aspects are covered, even with the inclusion of blockchain capabilities.

Due to the need to manage multiple devices connected to the Smart Grid, Shahryari and Anvari-Moghaddam [43] included bi-directional information and energy flow to facilitate the development of smart energy networks. In their proposal, they address the problems associated with managing a large number of connected devices and the massive volume of data generated by them, as well as the problems related to the transmission, processing and storage of data. To overcome those challenges, they propose to process data at the edge, improving performance and reducing the volume of transmitted data. They solve this problem with a smart gateway between the edge and the cloud that manages the demand side dynamically. One of the weak points of this research is that it does not ensure the reliability of the data received from different sources. The proposal presented in this paper ensures the reliability of data through the use of blockchain.

Moreover, Al Faruque and Vatanparvar [44] affirm in their work that energy management is necessary to address generation and consumption control in micro-grids, in residential, industrial and commercial scenarios. In their work, they address the need to provide scalable solutions for more efficient management of IoT resources. In this regard, they present an open-source Edge Computing platform that facilitates the implementation of energy management services and personalized control services. Their platform has been applied in the domestic sphere and in a public administration. However, the platform does not provide the necessary security nor does it facilitate the inclusion of third-party technologies in an easy and transparent way. In this regard, CAFCLA made it possible to include third-party technologies while GECA implements a set of security features.

In addition, the article of Li et al. [45] addresses demand response (DR) management using IoT devices. This work looks specifically at the security component. To overcome the problems of fraud in energy networks, the authors propose an Edge Computing scheme for secure demand response (Fog computing enabled Secure Demand Response—FSDR). Nonetheless, their solution only addresses security algorithms and management and doesn’t take into account the implementation of blockchain as GECA does.

The research that have been mentioned in this section are only a small example of how Edge Computing helps solve DR model problems. All of them make evident the importance of moving processing and communication to the edge in order to ingest more data and achieve a higher quality of data, optimizing scalability, security and management of energy sources and consumption.

#### 2.1.1. Domestic Environment

The great proliferation of low cost IoT devices, as well as the implementation of smart meters have facilitated the emergence of numerous solutions that seek to make the use of energy in homes more efficient. In this respect, the availability of Edge Computing solutions to this problem becomes key due to the huge amount of heterogeneous data sources that have to be managed in such a scenario [27].

Among the multiple approaches that we have evaluated, Yassine et al. [13] discusses the requirements and components of an IoT system for information capturing in smart homes. Among its priorities is the identification of daily activities that allow for the optimization of available energy resources. In this sense, the solution offers an Edge Computing platform designed to implement multiple services. Although this approach is very interesting, the work is still in a preliminary state and has not yet covered some aspects such as the security inherent to platforms with technologies such as blockchain, or how the platform could be extrapolated to other areas such as the commercial sector. All these aspects are covered by the proposal described in this paper.

Xia et al. [46] proposes an energy management framework based on Edge Computing. The main focus of this research is the amortization of deployed IoT devices to obtain efficient energy savings. It proposes a strategy of programming the optimum operating time for each device, taking into account the user’s preferences. Despite its good results, its solution only covers this specific case of use, making it difficult to extrapolate it to other energy efficiency environments. In this regard, the implementation by means of the CAFCLA framework in our proposal facilitates the design and application of similar solutions across different scenarios.

On the other hand, Chakraborty and Datta [47] proposes a home automation solution through the use of Edge Computing, Virtual IoT Devices and IoT. Their solution addresses very important issues in this area, such as the existence of multiple IoT standards, the heterogeneity of IoT devices and non-interoperability of existing solutions. They present a smart home architecture that covers these issues and how it might be implemented. However, there are no specific sensor implementations designated for increasing energy efficiency.

In addition, Zhou and Zhang [15] proposes a system based on Edge Computing for electrical demand forecasting in smart homes. Their system uses an intelligent edge node that stores heterogeneous data, although processing and analysis are done in the cloud. In addition, the system collects environmental data from other IoT devices, demonstrating a better quality of service and greater scalability of computing resources thanks to the edge-computing approach. However, this approach is not considered from the perspective of users and does not mention the benefits that users enjoy in the different scenarios. This is another element that we have achieved in our work by means of CAFCLA.

#### 2.1.2. Commercial and Public Building Environments

Commercial and public building environments are a sector in which energy efficiency has become necessary. Carbon emissions reduction and the price of energy are two of the main factors that cause companies and administrations to opt for the use of technology to reduce their energy consumption. As on previous occasions, the approaches to solve this problem are numerous. Below, we briefly describe some of the most relevant ones, which highlight the importance of having complete Edge Computing architectures for optimal deployment and full use of the technology.

García et al. [9] uses CAFCLA in order to improve energy efficiency in public buildings. They use this framework to deploy a serious game for increased energy savings. The game integrates multiple technologies that provide context-awareness and social computing capabilities. It includes IoT devices and real-time location identification, measuring and targeting social interactions. Although this is a novel approach and uses IoT technology to ingest information, most of the processing is done in a centralized server. In addition, it does not cover security issues or compare total savings with other buildings or solutions. This paper improves CAFCLA by combining it with the GECA architecture.

Fotopoulou et al. [48] considers that the deployment of innovative solutions in smart buildings allows for achieving significant reductions in energy consumption. For this, the adoption of energy efficiency techniques and the active participation of the occupants is necessary. Their solution presents an ecosystem with energy awareness that allows for the development of customized energy management services. The combination of IoT and Edge Computing techniques allows them to present an open and extensible architecture capable of exploiting in a homogeneous, efficient and scalable way a large amount of energy, environmental and behavioural data. The present paper improves this approach by, for example, including blockchain technologies for their implementation in real-time locating systems.

In [49], the authors develop a solution that implements IoT devices and machine learning algorithms to create a predictive model for forecast indoor temperature in smart buildings. Since temperature control is key in the conservation of energy in buildings, the work proposes Edge Computing as the premises on which the system is developed. Although this solution potentially makes it possible to achieve energy efficiency in buildings management, it does not define or implement the edge architecture, an aspect that is covered in this work through the integration of GECA and all its features.

One of the works evaluated is that presented by Khanna et al. [50]. The authors describe several architectures and frameworks that facilitate the development of intelligent structures for Smart Buildings. Their work highlights the importance of IoT architectures in the conservation of energy. This work doesn’t present a solution as such, but its conclusions show that architectures such as GECA are of vital importance in Smart Buildings environments.

Finally, Ferrández-Pastor et al. [51] presents an Edge Computing solution that integrates IoT devices with SoC (System on a Chip) capacity for the design of smart buildings. The authors consider the integration of different systems, such as monitoring energy consumption, temperature control, safety, comfort or operating costs, to develop smart facilities. Their solution overcomes some drawbacks of existing designs such as interoperability and scalability. The proposed architecture is experimental and is based on embedded devices. However, IoT approaches should be more flexible in terms of integration of components, in a similar way as CAFCLA and GECA are implemented.

## 3. Applying GECA to Provide Edge Computing and Blockchain Features to the CAFCLA Framework

**Figure 2:**
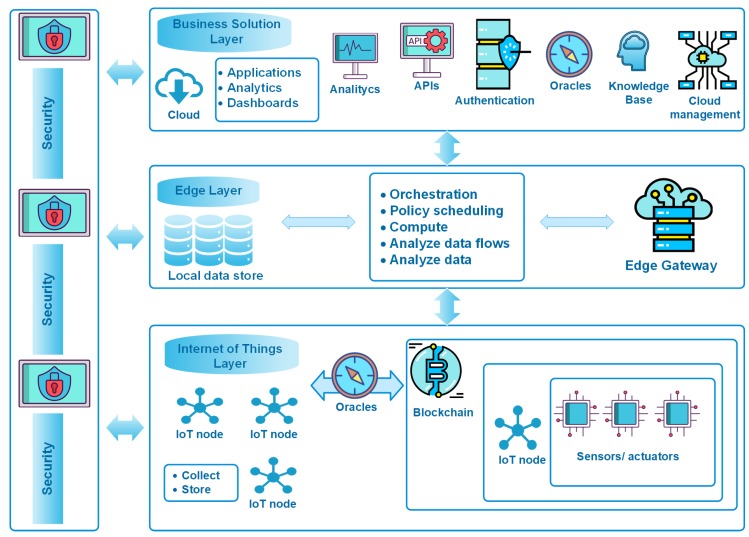
The Global Edge Computing Architecture (based on Sittón-Candanedo et al. [52]).

In [52], authors present GECA, a Global Edge Computing Architecture which integrates blockchain technologies to strengthen security on the three levels of architecture depicted in Figure 2.

The layers of GECA are described as follows:

- **IoT layer**: This layer is integrated by IoT devices such as sensors, actuators, controllers that are used to monitor equipment in operation, services or human activities. The layer uses a wireless standard such as Wi-Fi or ZigBee [53]. The creators of GECA incorporate blockchain technology and the concept of oracles in this layer. The oracles are responsible for verifying and sending data to the blockchain and work as intermediaries between the blockchain and the devices that generate data sets with values for temperature, humidity, market prices, payments, heart rate, movement, solar radiation and more. The generated data are regulated by microcontrollers with few computer resources and reduced storage capacity and must be sent to the Edge layer, which has computing modules with filtering and pre-processing capabilities. Thanks to the Crypto-IoT boards developed by the BISITE Research Group of the University of Salamanca (Spain), this layer complements the security of the architecture. Each board is formed by a USB module in host mode for connection to micro servers such as Raspberry Pi or neural processing modules, I2C sensors in plug and play modes, an ATSHA204A encryption chip with SHA-256 technology and XBee technology to support different telecommunication standards such as: ZigBee, Wi-Fi, LoRa, FM Radio, RPMA or Bluetooth [52]. Thanks to these data-agnostic electronic devices, it is possible to implement platforms and systems in which the integrity of the data collected by the different sensors is guaranteed following the blockchained IoT paradigm. This ensures that no data are modified by any other element of the network or that spurious data are injected into it. In this way, neither the origin nor the value of the data can be repudiated by any other observer component of the data. For these reasons, it is possible to implement smart contracts that are automatically executed if the agreed conditions are met and in which the nodes that sign these contracts cannot repudiate their compliance [54].
- **Edge layer**: This is the central layer of the architecture and it is in charge of the orchestration, monitoring and updating of the technological resources needed for the management of the organization’s activities. The Edge layer filters and pre-processes the data generated in the IoT layer in real-time, sending to the cloud the data used by Business Intelligence applications. The elements in charge of pre-processing are low-cost solutions, such as microcomputers based on educational architectures (such as Raspberry Pi or open architectures such as Orange Pi based on Raspberry Pi with the ability to work with Linux), with RAM between 256 MB and 1 GB as well as microprocessors from one to four cores with 1 GHz and the ability to add several SD cards with GB for storage. Likewise, if the organization requires greater computing capacity or online data availability, this layer supports edge gateways that are computers with greater capacity that function as an intersection between the devices of the IoT layer and the cloud. To manage the data in these IoT scenarios (smart cities, smart energy, industry 4.0, etc.), routers with sufficient processing power are incorporated into this layer. In addition, the edge gateways offer different interfaces for communication standards and cable or radio frequency transmission technologies, such as Ethernet, Wi-Fi, WLAN, Bluetooth, 3G mobile telephony, LTE, Zigbee, Z-Wave, CAN Bus, Modbus, BACnet or SCADA.
- **Business solution layer**: A set of services and business applications makes up this layer. Interactive interfaces are a part of the business application ecosystem; they are used to provide a more complex set of features. Furthermore, public cloud-based services can be used at this level of operation. The layer is made up of components that provide services to the different operating units of the business:
  - Analysis: Provides the capacity for data analysis and visualization, through case base reasoning and other automatic learning techniques.
  - Cloud Management: A storage and management service which segregates data physically or virtually, according to the tenant (department or work group). Scalability is viewed as the demand for infrastructure and resource growth.
  - Authentication: Effective through authorization and distributed transaction. Through the use of smart contracts in the public domain. In the private sphere, a permissioned blockchain is used.
  - Knowledge Base: A social machine is developed, it will allow for provision, supervise and update the connected technological resources.
  - APIs: These make it possible to call cloud services, so that they are available through a web browser or some other client application (standard methods such as: HTTP, RESTful, XML and SOAP).

After reviewing the state of the art in Section 2 and the principal features of GECA, the authors propose the integration of the design rules and features of this architecture into systems based on the CAFCLA framework. The aim of this is to provide edge node management and security, improving energy efficiency in public buildings. García et al. [55] and García et al. [56] define CAFCLA as a framework that allows for developing e-Learning applications and systems based on a pedagogical Computer Supported Collaborative Learning (CSCL) approach as well as the Ambient Intelligence paradigm.

**Figure 3:**
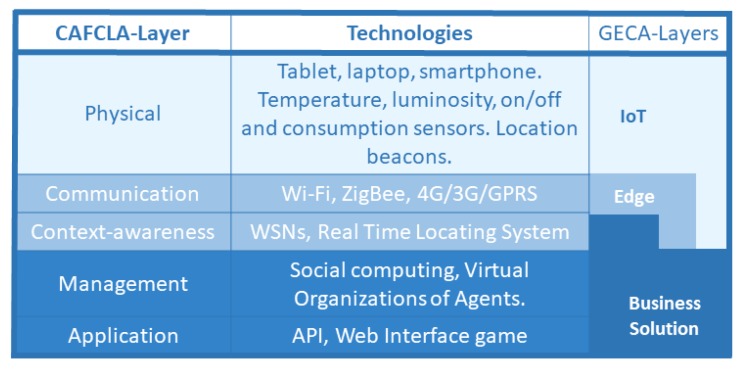
CAFCLA-GECA layers.

Figure 3 shows how the three layers of GECA can be implemented into systems based on the CAFCLA framework. CAFCLA has five layers that are described in depth by García et al. [55], (Physical, Communication, Context-awareness, Management and Application are modified and distributed according to GECA rules.

**Figure 4:**
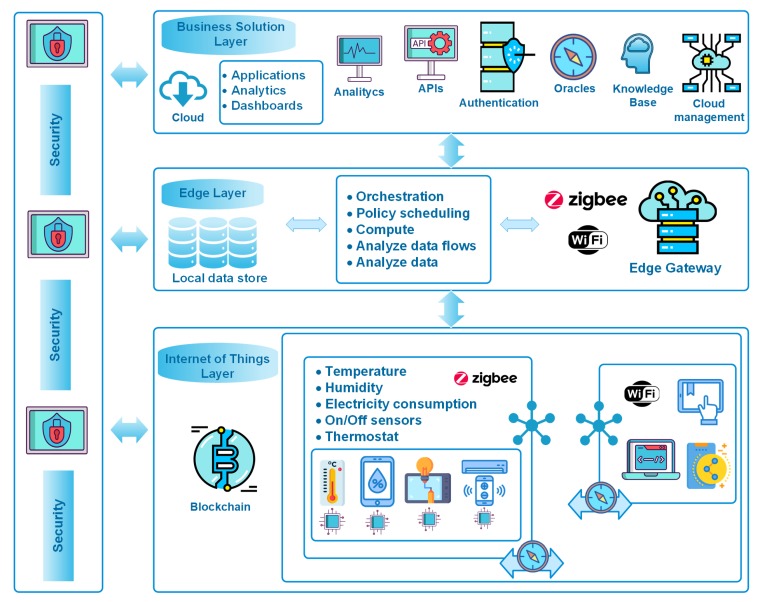
Applying the Global Edge Computing Architecture to Energy Efficiency scenarios.

In Figure 4, authors integrate the components of the physical layer of CAFCLA (i.e., temperature, humidity and on/off sensors, tablets, smartphones and others) in the IoT layer of GECA. Communications and Context-awareness layers and their elements, such as ZigBee and Wi-Fi, are included in IoT and Edge Layers. Likewise, Management and Application are incorporated in the Business Solution Layer.

In this sense, García et al. [24] propose the implementation of a system aimed at optimizing energy efficiency in public buildings. This serious game is based on the CAFCLA framework. Its participants are workers who are rewarded if they reduce their energy consumption. To this end, the system exploits the Context Awareness Layer of CAFCLA, continuously monitoring how people use energy during their working day in terms of lighting and Heating, Ventilation and Air Conditioning systems. It monitors the real-time location of workers, their activity, as well as the existing temperature and lighting in the workplace according to the external weather conditions. The advantage of this system is reduced data traffic between the IoT-Edge and Cloud layers thanks to the application of the design rules and features of the Global Edge Computing Architecture, evaluated in the next Section 4.

## 4. Use Case: Edge Computing and Social Computing for Energy Efficiency in Public Buildings

To evaluate and validate the benefits of including Edge Computing in applications based on IoT and Social Computing, we have conducted a case study. More specifically, this case study examines how the performance of the CAFCLA framework improves through the addition of an Edge Layer, following the Global Edge Computing Architecture. The enhanced version of the framework has been tested in a scenario aimed at identifying the efficiency of energy consumption in public buildings by means of Social Computing and Context-aware technologies. The former non-Edge version of CAFCLA had previously been tested and described by García et al. [24]. In this new work, the independent performance of the CAFCLA framework is assessed. Then, the performance of CAFCLA is measured when it operates under the design rules and features of the Global Edge Computing Architecture.

### 4.1. Evaluation Scenario

For this research, it is tested and compared the two versions of the system (previous non-Edge and new Edge-based) in the same scenario García et al. [24]. As in the previous work, the main objective of the scenario is the application of the IoT and Social Computing features of CAFCLA for the development of serious games that use contextual information and encourage a change in behaviour towards more efficient energy habits in public buildings and/or in the workplace.

The required contextual information is collected by means of IoT devices (forming a Wireless Sensor Network) that facilitate the acquisition of physical quantities associated with energy consumption (e.g., temperature, lightness) at different points, as well as information about the presence of each worker throughout the building (i.e., each worker’s location is detected by means of a Real-Time Locating System [57]), and focusing on an efficient use of lighting, workstations, Heating, Ventilation and Air Conditioning systems, as well as elevators. Furthermore, a Virtual Organization of Agents (VOA) [58] supports a serious game designed to make users more aware and help them acquire good habits in a natural way, encouraging a change in their behaviour, so that energy savings are achieved through a more efficient use of resources.

**Figure 5:**
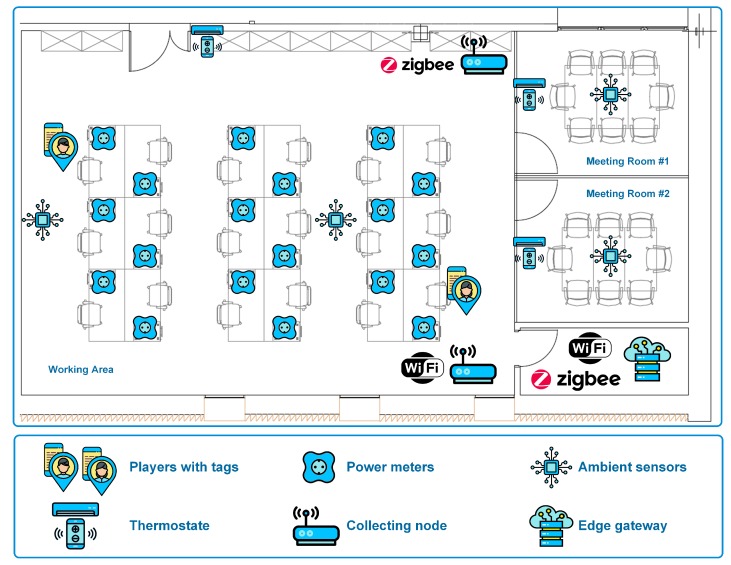
IoT devices and Edge Layer in the evaluation scenario for energy efficiency in public buildings (based on García et al. [24]).

Figure 5 depicts the laboratory of the BISITE Research Group (University of Salamanca, Spain) chosen as the implementation scenario. As can be seen, there is a main workspace (88 m${}^{2}$), with 18 desktops for 18 workers, all of them involved in the experiment, and two additional meeting rooms (12 m${}^{2}$ each one). This laboratory is located on the 2nd floor of the building. Therefore, workers must access it walking up the stairs or taking an elevator.

As described above, the CAFCLA framework defines its layers in terms of Physical, Communication, Contextual information, Management and Application layers [59], while the GECA architecture is formed by IoT, Edge and Business Solution layers [52], and the correspondence between them is reflected in Figure 3.

From the point of view of CAFCLA layers, the evaluation scenario can be described as follows:

- The Physical layer contains all the devices that will be used within the framework, which are depicted in Figure 5:
  - 1x Wi-Fi access point to provide each player with an Internet connection for their smartphone or laptop.
  - Some heterogeneous wireless sensor networks that gather the required contextual information: ambient temperature, lights switch on/off, brightness, energy consumption at each workstation, as well as location beacons to track workers throughout the environment:
    * 4x n-Core Sirius RadIOn devices with n-Core Sirius IOn-E expansion boards [24] (labeled as Ambient sensors on Figure 5), including a temperature and relative humidity sensor, a light sensor, and transmitting them every 60 s at maximum rate through ZigBee.
    * 9x n-Core Sirius RadIOn devices near elevators and two stair groups at each one of the three floors of the monitored building to detect if each worker used the stairs or the elevator to get to the lab and back.
    * 18x n-Core Sirius Quantum devices [60] acting as ZigBee tags that are worn by each worker to show their real-time location in the system.
    * 18x Cloogy devices [61], one for each workstation, a power consumption sensor transmitting measurements every 15 min via ZigBee.
  - 1x ZigBee collecting node to gather data coming from ZigBee devices.
  - 1x IoT-Edge gateway placed at computer office to collect data coming from the sensor nodes in the environment as well as the collecting node for the Real-Time Locating System. This IoT-Edge gateway collects ZigBee frames coming from sensor nodes and forwards data to the Business Solution Layer in the Cloud using an Internet connection.
- The Communication layer establishes the ZigBee protocol through which information will be transmitted between sensors and beacons. Wi-Fi transmits the information to the server and the devices of the participants.
- The Contextual information layer integrates the location engine and all the logic needed to perform effective data collection through the IoT devices forming the Physical layer. Furthermore, in this scenario, this layer exploits the features of the n-Core platform [60] and the n-Core Polaris Real-Time Locating System [62] to provide the system with sensing and locating capabilities.
- The Management layer includes a social machine, as well as all the logic and intelligence that allows for the management of the serious game.
- Finally, the Application layer develops the interaction interface for both game configuration and development.

Thanks to the use of Social Computing [63], the serious game provides the players with recommendations (in the form of daily emails) based on the most energy-efficient actions that have been taken by other workers. The nearest k-next-neighbor algorithm (kNN—k-Nearest Neighbors) [64] is used to determine different groups of workers, according to how each worker uses the lights, stairs/elevator, HVAC systems or the amount of energy they consume at their workstation.

If the worker completes a recommended action, they win 10 virtual coins, otherwise they are penalized and 10 virtual coins are taken away from them. To encourage participants, once they have collected 250 virtual coins, they can exchange them for something from the vending machine of the building. The actions that help to win or lose virtual coins are as follows:

- Avoid switching on lamps when natural lighting is above 200 lx (i.e., lumen/m${}^{2}$).
- Do not use the air conditioning system when the temperature is above 18 ${}^{\circ }$C in winter or under 25 ${}^{\circ }$C in summer.
- Obtain a daily power consumption below the average of the previous day.
- Use the stairs instead of the elevator.
- Turn off the lights and the air conditioning system when the last user leaves the lab.
- Belong to the group whose behaviour is efficient over a two-day period.

**Figure 6:**
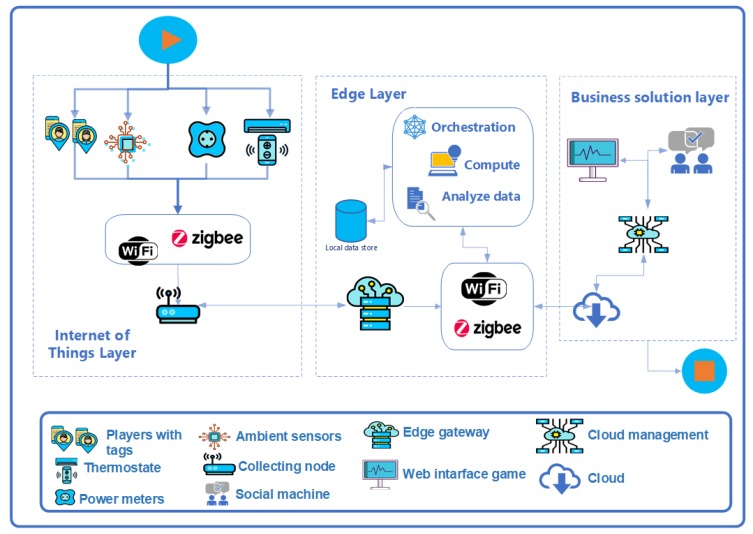
Data-flow chart of the evaluation scenario.

García et al. [24] concluded that gaming was a powerful tool for promoting energy-saving habits and also healthy behaviors, by means of encouraging workers to achieve higher scores during the performance of the game. Nonetheless, the objective of this new research is different. In this sense, Figure 6 presents the data-flow chart of the evaluation scenario, in which a new version of the system based on the CAFCLA framework would benefit from the application of the GECA architecture design guidelines. The benefits, thanks to the inclusion of the Edge Layer, include achieving reduced data traffic between the sensor networks and the Cloud.

### 4.2. Experimentation

Cloud providers such as Amazon Web Services, Microsoft Azure or Google Cloud offer pricing plans according to the amount of resources customers use over time [65]. Cloud pricing plans are quite complex to describe, but, at a high level, cloud providers charge for computing resources (use of CPU in terms of cores×GHz×s, as well as RAM in terms of GB×s), storage and database resources (in terms of GB×month, as well as total read and write operations per month), as well as network resources (in terms of amount of incoming and outgoing traffic), among others. The application of the Edge Computing paradigm can reduce the traffic that moves into and out of the Cloud and the amount of data that the Cloud stores, and the computing resources used up. To test and validate the benefits of using an Edge Computing architecture in the CAFCLA framework (i.e., the Global Edge Computing Architecture that the platform follows) in terms of savings in incoming (from IoT devices or Edge nodes to Cloud) and outgoing (from Cloud to the IoT devices or Edge nodes) transmission costs, the platform was evaluated in the same use case in the BISITE Research Group environment in a two four-week (28 calendar days each one) period. These two tests were carried out in separate time periods. The first test was carried out from 3 September 2018 to 30 September 2018, both days included, and the second test was performed from 1 October 2018 to 28 October 2018, both days included. Therefore, the same number of working days and weekends were considered within the two tests.

The first test was used as a baseline period with no Edge Layer present, just IoT Layer (IoT devices and IoT-Cloud gateway) and Business Solution Layer in the Cloud. The second one was used to verify the possibility of optimizing the performance of the same system through the addition of the Edge layer by means of the full application of the Global Edge Computing Architecture, that is, with IoT Layer, Edge Layer and Business Solution Layer on Cloud present and running within the platform. The two tests were subjected to exactly the same conditions in terms of IoT data sources.

In the first test, we measured the amount of data transferred in the old version of the system between the IoT sensors and the Cloud, both uplink traffic (from the IoT devices to the Cloud) and the downlink traffic (from the Cloud to the IoT devices). This version did not benefit from the advantages provided by the Edge Layer of the Global Edge Computing Architecture. In the second test, the same experiment was carried out to evaluate the new version of the CAFCLA-based energy efficiency system, which included the new Edge Layer of GECA. In this research, the new Crypto-IoT boards were not included in order to start the second test as soon as possible. Starting the test later would imply greater variations in daily sun hours and weather conditions and thus the inability to compare the results accurately). Otherwise, the use of lighting and HVAC by workers would be probably quite different in the second test with respect to the first test considered as baseline. Therefore, in the second test data, transmission was measured in two links: uplink and downlink traffic between the IoT sensors and the Edge layer (from IoT devices to Edge nodes, and from Edge nodes to IoT devices, respectively) and uplink and downlink traffic between the Edge layer and the Cloud (from the Edge nodes to the Cloud, and from the Cloud to the Edge nodes, respectively).

As mentioned previously, the system deployed in the first test was a non-Edge version; it did not include the Edge elements based on the Raspberry Pi 3 Model B and TensorFlowLite or blockchain. In this sense, in the first test, the IoT-Cloud gateways gathered the data through ZigBee and Wi-Fi and forwarded them directly to the Cloud by means of a fiber optics Internet connection without filtering, pre-processing, or encrypting them. On the contrary, in the second test, the new Edge elements based on the Raspberry Pi 3 Model B and TensorFlowLite were incorporated, thus following the full features and design rules of the Global Edge Computing Architecture. In both tests, the number of IoT data sources and the number of players were the same.

The Business Solution Layer of the energy efficiency system was deployed in Google Cloud using the App Engine as PaaS (Platform as a Service) to provide the backend of the system, including the Real-Time Locating System [62] (developed under .NET Core), the Google Cloud SQL to provide the relational database (configuration, users, node setup, etc.), Google Functions as FaaS (Function as a Service) developed under Node.js to provide the different REST APIs of the system and Google BigQuery to provide database for storing massive volumes of sensing data coming from IoT and Edge Layers. Furthermore, to develop the frontend, HTML5, Bootstrap 4 and Vue.js technologies were utilized.

### 4.3. Results

**Figure 7:**
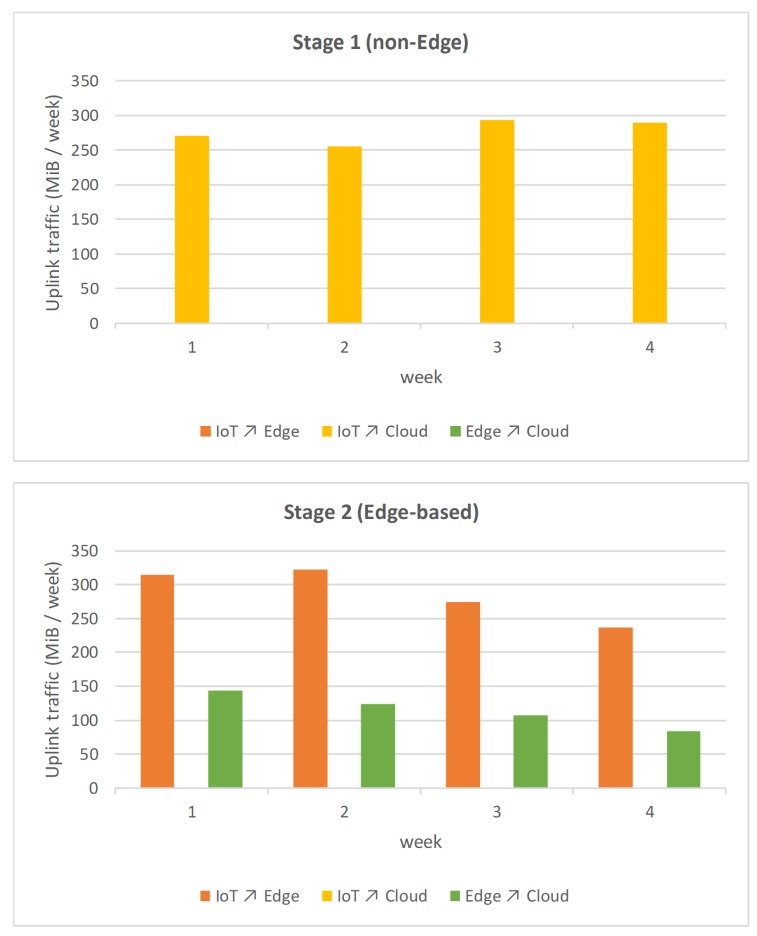
Comparison of the amount of data transferred in the two tests (uplink).

**Figure 8:**
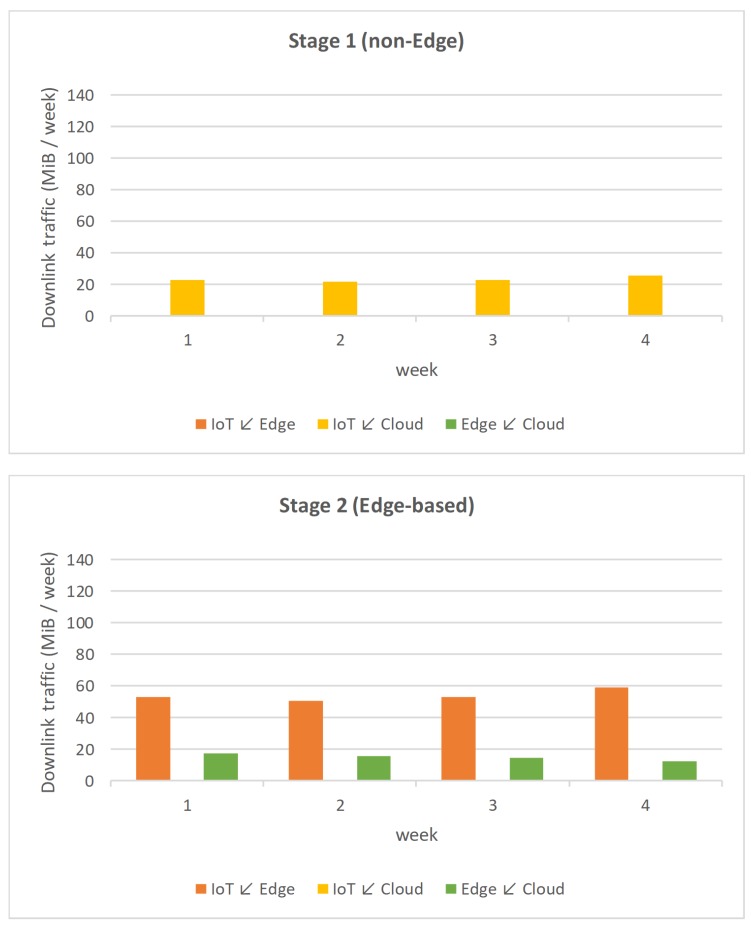
Comparison of the amount of data transferred in the two tests (downlink).

On the one hand, Figure 7 shows a graphical comparison between the uplink traffic (from IoT devices to Edge nodes, from IoT devices to Cloud and from Edge nodes to Cloud) in the two four-week tests, non-Edge and Edge-based, respectively. On the other hand, Figure 8 depicts the same comparison between the downlink traffic (from Edge nodes to IoT devices, from Cloud to IoT nodes and from Cloud to Edge nodes) in the two tests.

Logically, in Stage 1 (non-Edge), there is only a single direct bidirectional data flow between the IoT devices and the Cloud, since no Edge device is implemented at that stage. On the other hand, in Stage 2 (Edge-based), in which Edge devices are incorporated, there is no direct communication between IoT devices and the Cloud, but there are two bidirectional flows: the first between IoT devices and the Edge nodes, and the second between the Edge nodes and the Cloud.

Finally, Table 1 shows the total amount of data transferred uplink and downlink during the two four-week tests. The data in the table demonstrates that the application of the Global Edge Reference Architecture to the Social Computing energy efficiency system has been beneficial. It allowed for reducing the amount of total data transferred to the Cloud, thanks to the inclusion of the Edge layer. These results show that the GECA architecture has contributed considerably to our system, given that the same conditions had been established in the public building scenario and the level of sensitization was the same, 56.71% (58.47% in uplink and 36.00% in downlink).

In the usual cost scheme of Cloud providers, the storage and ingestion of data tends to have a reduced price, while the costs are higher both in the computation that is done on the data or the actual extraction of data out of the Cloud again [65]. Although the reduction in traffic is more significant in data uploading to the Cloud than in data downloading, reducing the traffic uploading to the Cloud allows for reducing the computing costs associated with the processing of such data. In this sense, thanks to the use of the GECA architecture in the new version of the platform for the implementation of the system, the reduction in the volume of data transferred to the necessary Cloud is much lower than in the first version of the system. This reduction could be different in other scenarios that would make possible the use of different filtering and pre-processing at the Edge Layer, such as smart farming scenarios as introduced by Sittón-Candanedo et al. [52].

In the scenario analyzed in this paper, the energy consumption reduction is much more significant than in Sittón-Candanedo et al. [52]. This is because in this case an indoor location system has been introduced; it sends much more data from the beacons of the real-time locating system running in the cloud. In this sense, the Edge layer is used in the Raspberry Pi 3 Model B, as an Edge gateway for filtering the duplicated frames coming from the ZigBee beacons through the collector node, so that the reduction of traffic that is uploaded to the Cloud is very noticeable. However, the reduction in traffic between the Edge and the Cloud could be even greater if the location engine were also implemented on the Edge, something that has not been done at this stage. Although the Raspberry Pi 3 model B is capable of running complex algorithms with a certain level of efficiency, it is necessary to adapt and optimize the n-Core locating engine so that its performance is adequate in microcomputers such as the Raspberry Pi 3 Model B.

## 5. Conclusions and Future Work

Nowadays, there are more and more applications in which it is necessary to obtain detailed information on different environmental and operational variables involved in a given environment. In this sense, technologies such as the Internet of Things, massive data repositories (Big Data) and data analysis techniques (Business Intelligence, Machine Learning) are presented as ideal tools for monitoring these variables, as well as the extraction of knowledge from them.

One of the application areas where these technologies can have a greater impact are Smart Energy and, more specifically, the reduction of energy consumption in public buildings. In this type of scenario, Cloud Computing technology makes it possible to always have up-to-date computer equipment and consume less energy as physical resources are being shared among many users by virtual machines. However, Cloud service providers charge for computing, storage, and network traffic services based on their use.

The results of this work demonstrate that it is possible to deploy systems that are more economic in terms of Cloud provider costs, by balancing the computation load from the Cloud to the Edge through devices with reduced computational capacities, consumption and hardware cost. These systems reduce costs because they do not transfer unnecessary information to the Cloud. This also means that computational and storage costs in the Cloud are lower.

In conclusion, this work has demonstrated the benefits of applying the design rules and the functionalities of the Global Edge Computing Architecture to the CAFCLA framework, especially in scenarios that involve the use of IoT and Social Computing technologies for greater energy efficiency. This has been the case with this new proposal, where an existing energy efficiency system has been improved notably with the application of CAFCLA and GECA, reducing the data traffic that enters the Cloud, especially lessening the amount of information that needs to be transmitted from the ZigBee beacons to the locating engine running in the Cloud.

The inclusion of the new Edge hardware (based on low-cost Raspberry Pi 3 Model B devices) makes sense to reduce the recurrent Cloud provider costs in terms of Cloud computing, storage and network resources. Moreover, the Edge layer allows for continuing to provide users with data analytics and not lose data if the communication with the Cloud is temporally interrupted. Furthermore, with this first step, we have set the basis for a further research on testing how this new Edge layer can provide new features such as Machine Learning techniques at the Edge (similarly to the RTLS engine).

However, we hypothesize that there is still a lot of room for improvement in the reduction of traffic that enters the Cloud from the Edge. This would be possible if a part of the engine were deployed to calculate the positions of users in the Edge itself. In this regard, a future work will consider deploying a part of the components of the locating engine in the Edge layer instead of the Business Solution layer. Once this development has been completed, new tests will be carried out in the same scenario to compare the potential improvement.

Furthermore, new experiments will include a comparison not only in terms of the data transferred, but also in terms of data latency between sensors and users, as well as computing, storage and network costs over a chosen Cloud provider. Likewise, in the future, it will be advanced even further on the integration of CAFCLA and GECA in relation to the implementation of Crypto-IoT boards with IoT sensitization and location devices, thus improving the system in terms of security, traceability and data integrity.

## Figures and Tables

**Table 1:** Data transmitted between the different IoT, Edge and Cloud components in the two four-week tests.

Stage	Stage 1 (non-Edge)	Stage 2 (Edge-based)
**IoT devices ↗ Edge nodes**	0 MiB	1148.867 MiB
**IoT devices ↙ Edge nodes**	0 MiB	215.861 MiB
**IoT devices ↗ Cloud**	1107.643 MiB	0 MiB
**IoT devices ↙ Cloud**	93.757 MiB	0 MiB
**Edge nodes ↗ Cloud**	0 MiB	460.037 MiB
**Edge nodes ↙ Cloud**	0 MiB	59.996 MiB
